# Total and Free Serum Sialic Acid Concentration in Liver Diseases

**DOI:** 10.1155/2014/876096

**Published:** 2014-05-18

**Authors:** Ewa Gruszewska, Bogdan Cylwik, Anatol Panasiuk, Maciej Szmitkowski, Robert Flisiak, Lech Chrostek

**Affiliations:** ^1^Department of Biochemical Diagnostics, Medical University of Bialystok, Waszyngtona 15A Street, 15-269 Bialystok, Poland; ^2^Department of Pediatric Laboratory Diagnostics, Medical University of Bialystok, Waszyngtona 17 Street, 15-269 Bialystok, Poland; ^3^Department of Infectious Diseases and Hepatology, Medical University of Bialystok, Zurawia 15 Street, 15-540 Bialystok, Poland

## Abstract

*Background*. The objective of this study was to compare the levels of total (TSA) and free (FSA) sialic acid in acute and chronic liver diseases. *Materials and Methods*. The serum TSA and FSA levels were determined in 278 patients suffering from acute and chronic liver diseases of different etiologies. TSA was estimated by enzymatic method and FSA by the thiobarbituric method modified by Skoza and Mohos. *Results*. There were no significant differences in the serum TSA concentration between liver diseases of different etiologies, although in most of the liver diseases the mean TSA level was significantly lower than that in the control group. In contrast to TSA, the concentration of FSA appears to differ between liver diseases. In toxic hepatitis it was higher than that in nonalcoholic cirrhosis. However, neither of them differs between alcoholic and nonalcoholic cirrhosis or between liver tumors and tumors with cirrhosis. *Conclusions*. We conclude that the changes in concentrations of TSA and FSA during the same liver diseases indicate significant disturbances in sialylation of serum glycoproteins.

## 1. Introduction

Most of the serum proteins are glycoproteins, in which glycans are terminated with sialic acid residues [1–3]. In liver diseases, the changes in the concentration of sialylated glycoproteins in the blood have been reported [4, 5]. These changes should affect the concentration of total sialic acid (TSA). On the other hand, it is well known that the changes in protein glycosylation play an important role in the pathogenesis and progression of liver diseases [1, 2, 6, 7]. Because the most common disturbances in glycosylation rely on the increase of enzymatic activity that cuts off sialic acid residues from serum glycoproteins and/or on the decrease of enzymatic activity that binds sugar residues to oligosaccharide chains, these processes may result in increase of free sialic acid (FSA) concentration in the blood [6, 8].

The aim of this study was to assess the changes in the sialylation of serum glycoproteins by measuring total (TSA) and free sialic acid (FSA) concentration in the sera of patients suffering from acute and chronic liver diseases.

## 2. Materials and Methods

### 2.1. Subjects

The tested group consisted of 278 patients (99 females and 179 males) who were admitted to the Department of Infectious Diseases and Hepatology of University Hospital of Bialystok. The patients were divided into subgroups according to the diagnosis of liver diseases: 54 had alcoholic cirrhosis (AC), 34 nonalcoholic cirrhosis (NAC), 23 chronic nonviral hepatitis (CH), 32 toxic hepatitis (TH), 20 chronic viral hepatitis C (HCV), 17 chronic viral hepatitis B (HBV), 14 autoimmune hepatitis (AIH), 15 acute hepatitis B (AHB), 16 primary biliary cirrhosis (PBC), 14 fatty liver (FL), 24 primary liver cancer (HCC), and 15 primary liver cancer and cirrhosis (HCC + C). The diagnosis was performed on the basis of signs and symptoms of the disease, physical and clinical exam (ultrasonography and fine-needle biopsy in justified cases), and biochemical liver panel known as liver function tests (AST, ALT, and GGT). The diagnosis of viral hepatitis was supported by serological tests (HBsAg and anti-HCV). The causes of nonalcoholic liver cirrhosis were as follows: HBV-14, HCV-9, and unidentified factors-12. The toxic hepatitis was caused by alcohol abuse in 22 cases and drugs abuse in 10 cases. To confirm the diagnosis of primary biliary cirrhosis we performed the mitochondrial antibody test (AMA).

### 2.2. Controls

The control group consisted of 50 healthy subjects (18 females and 32 males) recruited from hospital workers. All subjects (healthy and sick) gave their consent to participate in the studies. The study was approved by the Bioethical Committee of the Medical University of Bialystok.

### 2.3. Sample Collection

Blood samples were taken by peripheral vein puncture once after admission and before treatment. The sera were separated by centrifugation at 1500 ×g for 10 min at room temperature and stored at −86°C until analysis.

### 2.4. TSA Assay

TSA concentration in the serum was measured on the Microplate Fluorescence Reader FL600 (Bio-Tek, USA) according to the enzymatic method (EnzyChrom Sialic Acid Assay Kit, BioAssay System, Hayward, USA) using the colorimetric procedure. Each determination in a single sample was performed three times. The samples should be pretreated in hydrolysis procedure, in which neuraminidase released the N-acetylneuraminic acid (NANA) from glycolinkages. In the next step the NANA is decomposed into N-acetylmannosamine and pyruvic acid in the presence of aldolase. Then the pyruvate is oxidized by pyruvate oxidase to acetyl phosphate, carbon dioxide, and hydrogen peroxide. In the last step, peroxidases and hydrogen peroxide convert 4-aminoantipyrine and N-ethyl-N-2-hydroxyethyl-3-toluidine to red coloured derivative. Briefly, to 20 *μ*L of serum was added 80 *μ*L of hydrolyzing reagent. This mixture was incubated in a water bath at 80°C for 60 minutes. After that the tubes were cooled to room temperature. Next, 20 *μ*L of neutralizing reagent was added to the reaction mixtures and the tubes were centrifuged for 5 minutes at 14,000 rpm at room temperature. After that, the samples were applied to a 96-well microplate in the ratio 10 *μ*L of sample and 90 *μ*L of working reagent, which includes N-acetylneuraminic acid aldolase, pyruvate oxidase, hydrogen peroxide, colored indicator, and a buffer. The microplate was incubated at room temperature for 60 minutes. The colour intensity of the reaction product at 570 nm is directly proportional to sialic acid concentration in the sample. The TSA concentration was read from standard curve (0; 0.3; 0.6; and 1.0 mM/L stock solution).

### 2.5. FSA Assay

FSA was determined using the thiobarbituric method of Skoza and Mohos [9]. Each determination in a single sample was performed three times. All reagents were from Sigma-Aldrich Chemie GmbH. Briefly, to 100 *μ*L of serum was added 250 *μ*L of periodic reagent (0.025 N periodic acid in 0.125 N sulfuric acid) and then it was mixed and incubated for 30 minutes at 37°C. The incubation was stopped by adding 200 *μ*L of the sodium arsenite (2% sodium arsenite in 0.22 M hydrochloric acid). After disappearance of yellow color derived from the released iodine, the 1.5 mL of thiobarbituric acid (0.1 M, pH 9.0) was added to a reaction mixture. Next, tubes were placed in boiling water for 7.5 minutes. Immediately after that, the tubes were transferred to an ice bath and left for 10 minutes. To each tube 1.5 mL of sulfoxide dimethyl was added. Measurements were performed at 549 nm using quartz cuvettes on the spectrophotometer Shimadzu UV-1202 (Shimadzu Europa GmbH, Duisburg, Germany). The amount of FSA was calculated from the following equation:

(1) μmol  of  FSA=V×OD54968=3.55×OD54968=0.0522×OD549,

where *V* is the final volume of the solution and OD_549_ is the optical density at 549 nm [10].

### 2.6. Other Laboratory Assays

For characteristics of patients the following tests were performed: alanine aminotransferase (ALT), aspartate aminotransferase (AST), gamma glutamyltransferase (GGT), carbohydrate-deficient transferrin (CDT), prothrombin time (PT), and mean corpuscular volume (MCV). Almost all of biochemical tests (ALT, AST, and GGT) were done on the Architect c8000 system (Abbott Laboratories, Abbott Park, IL, USA) using the kits from Abbott Diagnostics (Wiesbaden, Germany). The CDT values were assayed by immunonephelometry using N-Latex CDT test (Siemens Healthcare Diagnostics, Marburg, Germany) on BN II System (Siemens Healthcare Diagnostics, USA). MCV was measured using a hematological analyzer ADVIA 120 (Bayer, Tarrytown, USA) and prothrombin time on the STA Compact Hemostasis Analyzer (Diagnostica Stago, France).

### 2.7. Statistical Analysis

Significance of differences between groups (tested and control) was evaluated by Mann-Whitney* U *test. To test the hypothesis about differences in concentration of TSA and FSA in liver diseases of different etiology, ANOVA rank Kruskal-Wallis test was performed. Because the chance of finding one or more significant differences in 12 tested groups was 45.96% (Bonferroni correction factor), we performed the nonparametric multiple comparison test (post hoc test for Kruskal-Wallis) to ascertain which of the intermediate medians are significantly different. *P* < 0.05 was considered statistically significant.

## 3. Results

Table 1 presents laboratory tests performed for characteristics of patients with liver diseases. In most of liver diseases the mean values of MCV, PT, AST, ALT, and GGT were significantly higher than those in the control group. There were no significant differences in the serum values of  CDT  between liver diseases and control group.

**Figure 1:**
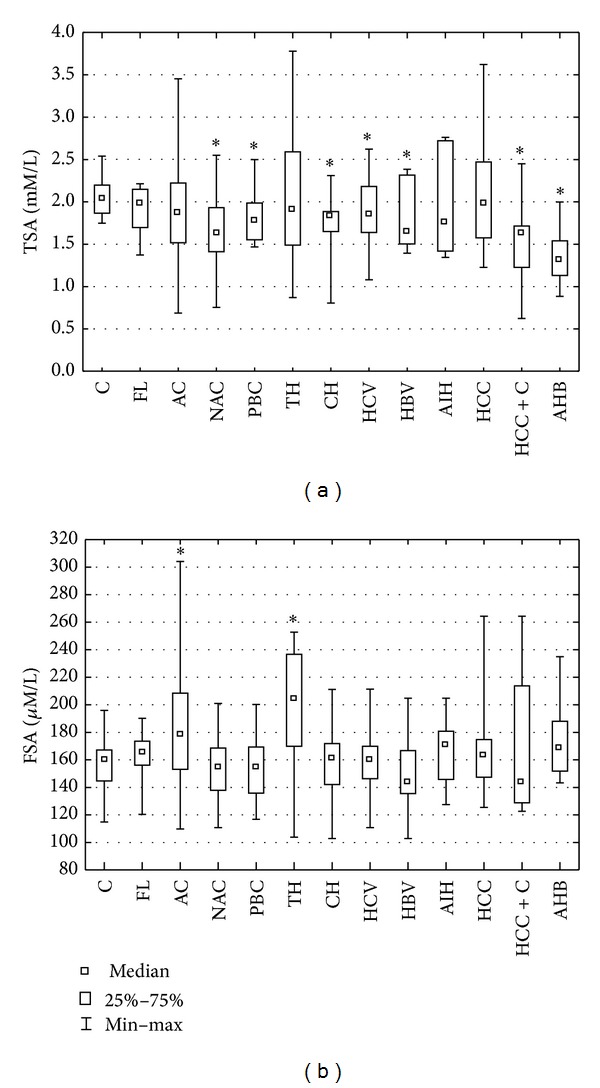
TSA (a) and FSA (b) concentrations in the sera of patients with liver diseases of different etiology. Results are presented as median and range. *Significant difference in comparison to controls. C: controls, FL: fatty liver, AC: alcoholic cirrhosis, NAC: nonalcoholic cirrhosis, PBC: primary biliary cirrhosis, TH: toxic hepatitis, CH: chronic nonviral hepatitis, HCV: chronic viral hepatitis C, HBV: chronic viral hepatitis B, AIH: autoimmune hepatitis, HCC: primary liver cancer, HCC + C: primary liver cancer and cirrhosis, and AHB: acute hepatitis B.

There were no significant differences in the serum TSA concentration between liver diseases of different etiologies (*P* = 0.143; ANOVA rank Kruskal-Wallis), but in most of the liver diseases the mean TSA levels were significantly different than in the control group. Further analysis revealed that the mean TSA concentration in nonalcoholic cirrhosis (NAC), primary biliary cirrhosis (PBC), chronic nonviral hepatitis (CH), chronic viral hepatitis C (HCV) and B (HBV), primary liver cancer and cirrhosis (HCC + C), and acute hepatitis B (AHB) was significantly decreased when compared with the control group (*P* < 0.001; *P* = 0.044; *P* < 0.001; *P* = 0.020; *P* = 0.024; *P* < 0.001; *P* < 0.001, resp.) (Figure 1(a)).

The mean serum concentration of FSA appears to be different between liver diseases of different etiologies (*P* = 0.015; ANOVA rank Kruskal-Wallis test). Post hoc analysis for Kruskal-Wallis test indicated that the mean value of FSA for the toxic hepatitis (TH) was significantly higher than the mean value for nonalcoholic cirrhosis (NAC) (*P* = 0.022). The serum concentration of FSA in patients with toxic hepatitis (TH) and in patients with alcoholic cirrhosis (AC) was significantly increased when compared to the control group (*P* < 0.001; *P* = 0.010, resp.) (Figure 1(b)).

## 4. Discussion

In our study we have measured the serum concentrations of TSA and FSA in liver diseases. Generally, we detected decreased levels of TSA in hepatitis of different etiology, cirrhosis, and liver cancer. Our results are similar to the results of Matsuzaki and coworkers which indicated that the serum level of TSA in patients with compensated cirrhosis was significantly lower than that in the control group and it was decreased further in those with decompensated cirrhosis [11]. The level of TSA in chronic hepatitis appeared to be similar to the level in the control group [11]. Though there were differences in TSA concentration in comparison to the controls, we did not find differences between liver diseases.

Interestingly, we did not observe the changes in TSA concentrations in patients with alcoholic cirrhosis. In our opinion, this fact may be the result of two opposing mechanisms. At first, the cirrhosis (nonalcoholic) causes the decrease of TSA concentration; secondly, alcohol abuse causes the increase of  TSA level [12–14]. The proof for the first argument may be the study of Stefenelli et al. who showed significantly lower TSA values in chronic liver diseases (among other cirrhoses) in comparison to malignant and noninflammatory diseases [15]. Matsuzaki et al. also point out that serum TSA concentrations in patients with compensated cirrhosis were significantly lower than in the control subjects and were decreased further in patients with decompensation [11]. In contrast, Arif and coworkers reported higher level of SA in advanced and terminal stages of disease but normal level in early ones [5]. They suggested that these results are related to aberration in carbohydrate structure of fibrinogen, which contains 0.6% of sialic acid, because both, fibrinogen and sialic acid, are the acute-phase reactants. These data confirmed that the unchanged concentration of total sialic acid in alcohol cirrhosis is the result of two factors: liver damage by cirrhosis and alcohol.

Similarly to the patients with nonalcoholic cirrhosis the total sialic concentration was also significantly decreased in patients suffering from liver cancer accompanied by cirrhosis in comparison to the control group. However, in patients with liver cancers without cirrhosis the TSA concentrations were slightly decreased, near normal. This can be explained by intensity of glycosylation disturbances during malignant diseases. There are many reports certifying the changes of sialic acid concentration in the course of malignant transformation [16, 17]. Besides, Stefenelli et al. confirmed that cases with extensive liver cirrhosis are characterised by continuous decline in the serum sialic acid level, especially in very severe cases with complications [15].

In our study we have also shown that FSA concentrations were significantly higher in toxic hepatitis than in nonalcoholic cirrhosis. This difference can be explained by the pathogenesis of toxic hepatitis due to excessive consumption of alcohol in about 70% of patients. We suggest that differences in FSA concentration between these diseases are the result of aberrant glycosylation in alcohol abusers. Additional confirmation of this hypothesis is the comparisons of FSA concentrations in patients with alcoholic and nonalcoholic cirrhosis. The concentration of FSA in alcoholic cirrhosis was significantly higher than in controls while in nonalcoholic cirrhosis the FSA level was the same as in the control group. Therefore, in the current literature there are reports showing the aberrations of glycosylations in liver diseases, but the exact mechanisms of these changes in each liver disease are not known or they are not clearly explained. In our study we tried to explain these mechanisms by the measurements of total and free serum sialic acid concentrations. Also, in the present literature there is no information about behavior of the FSA concentrations in nonalcoholic liver disease or whether there are differences in relation to alcoholic liver disease. We suggest that the FSA serum concentration can be useful in the differential diagnosis of  liver diseases, especially between toxic hepatitis and nonalcoholic cirrhosis. The causes of the changes in FSA concentration may be the alterations in glycosylation of glycoproteins in the liver diseases. These can rely on the increased desialylation, fucosylation, and branching and increased amounts of bisecting N-acetylglucosamine (GlcNAc) [6]. These changes may be explained by the alterations in the activity of enzymes, especially glycosyltransferases. These alterations occur in all liver diseases but with different intensities. The most important alterations observed in alcoholic liver disease are desialylation of transferrin and also haptoglobin, alpha 1-antitrypsin, and ceruloplasmin [6, 18]. In fatty liver diseases there is accumulation of apolipoprotein-B with increased amounts of bisecting GlcNAc and the increased accumulation of apolipoprotein A-1. In viral liver diseases there is increase in the fucosylation of alpha 1-antitrypsin, alpha 1-acid glycoprotein, and haptoglobin [6]. In liver cancer the majority of aberrations are the changes in the activity of N-acetylglucosaminyltransferase III (GnT-III) and N-acetylglucosaminyltransferase V (GnT-V), which respond to formation of branching and addition of bisecting GlcNAc [6].

In our previous study, we have described TSA and FSA in alcoholic and nonalcoholic cirrhosis and chronic viral hepatitis [19]. In the present study, we extended these subgroups and added other liver diseases, in particular, toxic hepatitis, liver cancers, and acute liver disease. This comparison more clearly revealed that the levels of TSA do not change between liver diseases, although almost in all cases they were lower than in healthy people. In the previous study TSA was diminished only in nonalcoholic cirrhosis [19]. However, the concentration of FSA was different not only between cirrhosis and chronic hepatitis but also between cirrhosis and toxic hepatitis. This suggests that in the course of most of the liver diseases the sialylation of proteins plays a significant role.

## 5. Conclusions

We suggest that the changes in the concentration of TSA and FSA in some liver diseases confirm the presence of significant aberrations in the sialylation of serum glycoproteins in these diseases.

## Figures and Tables

**Table 1:** The laboratory characteristics of patients with liver diseases and controls.

	MCV (fL)	PT (sec)	ALT (IU/L)	AST (IU/L)	GGT (IU/L)	CDT (mg/L)
C	88 80.2–94	12.45 11.7–13.8	16.5 8–39	23 14–39	21.5 8–46	43.3 26.8–61

FL	89.3 82–99	10.85* 10.2–15.6	75.5* 20–337	43* 29–136	63* 34–171	—

AC	98.2* 78–118.2	15* 7.2–31.7	32* 8–435	82* 23–1574	206* 12–2126	37.9 20.4–75.9

NAC	92* 61.1–108	14.45* 10.3–27.6	29.5* 6–115	46 13–146	58* 12–327	38.75 21.8–57.6

PBC	94* 86–105.8	14.2 9.9–20.3	31* 15–493	73* 35–245	142* 6–336	43.8 23.7–58.4

TH	99.1* 80.3–120	14.7 11–31.4	50* 7–302	60* 23–382	216* 9–4050	44.3 21.8–82.4

CH	89.6 77–97.7	12 9.8–17	72* 12–925	52* 16–674	44* 5–105	41.9 29.5–72.3

HCV	89.5* 85–102.1	12.2 10.1–15.5	60* 19–551	48* 19–236	75* 3–898	48.7 27.9–77.8

HBV	94.8* 84.9–97.4	13.1 11–14.5	62* 28–412	52* 27–378	53* 9–221	37.2 20.4–51.9

AIH	92* 87–94	13.2 9.7–20.6	141* 63–916	160* 58–460	232* 111–561	50.5 27–70.73

HCC	90.9 76–104.4	12.65 11–16.6	43* 6–194	84* 30–304	182* 81–1175	39.7 24.4–102

HCC + C	96.8* 85–98	17.6* 12.4–20	20.5 6–119	79* 35–195	141* 53–554	46 25–76.6

AHB	86.6 78.3–90	13.8 11.8–14.5	831.5* 726–1774	680.5* 125–1972	231* 168–453	—

Data are median and ranges. MCV: mean corpuscular volume, PT: prothrombin time, ALT: alanine aminotransferase, AST: aspartate aminotransferase, GGT: gamma glutamyltransferase, CDT: carbohydrate-deficient transferrin, C: controls, FL: fatty liver, AC: alcoholic cirrhosis, NAC: nonalcoholic cirrhosis, PBC: primary biliary cirrhosis, TH: toxic hepatitis, CH: chronic nonviral hepatitis, HCV: chronic viral hepatitis C, HBV: chronic viral hepatitis B, AIH: autoimmune hepatitis, HCC: primary liver cancer, HCC + C: primary liver cancer and cirrhosis, and AHB: acute hepatitis B.

*Significant differences in comparison to the control group.
